# Daily metabolic expenditures: estimates from US, UK and polish time-use data

**DOI:** 10.1186/s12889-019-6762-9

**Published:** 2019-06-03

**Authors:** Teresa Harms, David Berrigan, Jonathan Gershuny

**Affiliations:** 1 Department of Social Science, Centre for Time Use Research, University College London, 55–59 Gordon Square, London, WCH 0NU UK; 2 National Cancer Institute, Division of Cancer Control and Population Sciences, Bethesda, MD USA

**Keywords:** Time use diary, Time use survey, Physical activity, Physical activity energy expenditure, Metabolic equivalent of task, Ainsworth compendia, METs linkage

## Abstract

**Background:**

Behaviour has diverse economic, social and health consequences. Linking time spent in different daily activities to energy expenditure (EE) is one way of investigating the health and physiological consequences of behaviour and identifying targets to improve population health and well-being.

**Methods:**

We estimated behaviour-related EE for respondents to time use surveys (TUS) from three countries: UK 2001, Poland 2012 and US 2003–13. The Harmonised Multinational Time Use Survey (MTUS) activity categories were matched to MET estimates from the 2011 Compendium of Physical Activities. We attach METs values to each successive activity in the TUS, together with both the original UK, Polish and US activity classifications and the 68-category MTUS activity classification. We used TUS estimates of activity durations across 24-h to estimate the Physical Activity Level (PAL) for respondents from the three countries and the average time spent and MET values for different activity categories.

**Results:**

PAL values ranged from 1.59 in the US to 1.74 in Poland. The main sources of daily EE from PA were paid and unpaid work activities. Discretionary PA accounted for only a very small part (~ 3%) of adult daily energy expenditures. Using the harmonised MTUS 68-activity classification reduced the variability of the aggregate PAEE measure by ~ 20%, but the patterns of association between key demographics (age, sex, educational attainment) were unaffected. TUS data were further used to (1) identify sources of daily PA, and (2) assess adherence to physical activity guidelines (PAG) on a single-day basis. Estimated adherence levels were similar to those reported from other TUS as well as frequency based estimates.

**Conclusions:**

Comparative studies of energy expenditure based on harmonised time use activity categories could provide insight into the relative importance of different activities for energy expenditure across different countries and demographic groups. However, new observational studies combining TUS data with accelerometer, direct observation and other measures of activity intensity are required for more accurate MET assignments to activity categories in TUS.

**Electronic supplementary material:**

The online version of this article (10.1186/s12889-019-6762-9) contains supplementary material, which is available to authorized users.

## Background

Engaging in regular physical activity (PA) is now recognised as a critical target for the prevention of major non-communicable diseases [1]. Further progress in understanding the health consequences of patterns of inactivity and sedentary behaviour (SB), and in evaluating interventions and policies aimed at reducing these adverse effects, depends on adequate measurement of patterns of activity and inactivity throughout the day. An increasing number of studies report that maintaining health may depend on the allocation of time across the entire 24-h day, including sleep [2]. Additionally, recent epidemiological evidence has focused on the health benefits of intermittent and incidental daily activities (e.g. walking, light housework) and on the negative associations between health and sedentary time [3, 4].

Interest in capturing daily activities across the entire day and dealing with the measurement challenges associated with diverse and intermittent activities over the entire 24-h period have led to substantial research efforts aimed at improving the recording and measurement of PA [5–7]. These efforts have included a focus on direct (device-based) measurement [8–13] and self-report 24-h recall instruments [14, 15]. These measurement modalities attempt to address the cognitive challenges associated with summarising complex patterns of activity over longer time periods, and in the case of device-based measurement, the challenge of accurately assessing PA intensity [16, 17]. Concurrently, there has been an increased interest in the use of TUS data to examine PA [18–22].

TUS have been conducted for decades and used by sociologists and economists to inform diverse research topics. In the United States, the American Time Use Survey (ATUS), sponsored by the Bureau of Labor Statistics and conducted by the US Census Bureau as part of the Current Population Survey, has published time use data every year since 2004 [23]. The largest repository of time use data in Europe is the Multinational Time Use Study (MTUS) [24]. Established in the 1980s, it holds 60 harmonised time use datasets from 25 countries across Europe, North America, Asia and Oceania. An increasing number of academic and government researchers worldwide are collecting and analysing 24-h self-report time use diary (TUD) data because they generate representative and detailed national accounts of the time people spend in a wide range of everyday activities (e.g. paid and unpaid work, sleeping, leisure, eating, watching TV, playing sports). TUS also provide data on how daily life varies across demographic groups, countries and decades [25, 26].

Time use diaries, and the closely related 24-h PA recall method, have several advantages compared with standardised survey questions for estimating the frequency and duration of different daily behaviours [15, 27–30]. For most TUS, respondents report (in their own words) the activities in which they engaged; some surveys use ‘light diaries’ where diarists select from a list of specific activities for the current or previous day. Socio-demographic data are collected in all TUS and a number of recent studies also ask for self-reported height, weight and health status [31].

The continuous and sequential recording used in time use diaries covers all the activities in which respondents engage across 24-h, in contrast with the un-contextualised behaviour-specific approach of frequency or standardised survey questionnaires such as the International Physical Activity Questionnaire (IPAQ). TUD recording makes it difficult for respondents to manipulate subsequent activities (e.g. substituting watching TV for going to the gym), which lowers social desirability bias and measurement error [26, 32, 33] . TUDs also avoid many of the problems associated with questionnaire methods because they limit the recall period to no more than a day [20], although note that physical activity research has used 24-h, 3-day and 7-day PA recall instruments that adopt a diary like temporally sequenced approach [34, 35]. Responses to standardised PAQ questions are known to overestimate the frequency of many activities and this overestimation is particularly apparent for socially desirable activities such as PA and paid work [36].

In a small-scale validation study, van de Ploeg and colleagues [20] found relatively high correlations between PA inferred from TUS data and objectively-measured accelerometer data, concluding that time use data are more valid for non-occupational PA population monitoring than more traditional self-report methods (e.g. IPAQ). A growing number of validation studies have reported good performance of 24-h PA recalls [14] although biases can still be detected [15, 37]. Self-administered 24-h recalls could decrease costs and increase use of these methods in epidemiological and other health studies [38].

A major challenge in applying TUS data to research questions concerning PA involves understanding the nature of the activity categories used in TUS. As these surveys were designed by economists and sociologists, the categories reflect their interest in the economic and social functions of time. Therefore, activity categories from TUS often combine activities that include very different levels of EE, which can lead to misleading associations with certain types of daily activities and concomitant health outcomes [39]. For example, time use activity categories such as ‘sport’ may include both watching and participating and similarly, activity categories related to unpaid work (e.g. housework) might include a wide range of PA intensities [40–42]. Better understanding the utility of existing TUS for retrospective analyses of PA could emerge from efforts to validate estimates of time spent in specific activities reported in TUS (e.g. ‘swimming’) or overall PAEE estimates.

One approach to exploring the utility of TUS for PA research involves assigning MET (Metabolic Equivalent of Task) values to daily activities [39, 43, 44] using compendia of MET values for specific activities [22, 45, 46]. One early example of this approach involved the assignment of MET scores to activities in the National Human Activity Pattern Survey of the US Environmental Protection Agency [43]. This large study (~ 7500 adults) from 1992 to 1994 highlighted the largely sedentary nature of the US population by this time and as well as the key role of paid and unpaid work as sources of energy expenditure. Since then, several studies have examined time trends, international variation and gender differences in energy expenditure via linkage of MET scores to TUS including studies from the US, UK, Brazil, India and China [44], the American Heritage Time Use Study [47, 48] and the 2006 Australian Time Use Survey [49].

Tudor-Locke and colleagues [39, 50] describe important methodological work linking the 2003 ATUS Activity Coding Lexicon with the Ainsworth Compendium [51, 52] of MET scores to allow estimates of energy expenditure over the 24-h day. This work focused attention on the lack of detailed activity descriptions for time spent in paid and some types of unpaid work and provided a readily accessible online tabulation of activities and MET scores [53]. Together these studies support further efforts to link TUS derived activity patterns to measures of energy expenditure.

The central purpose of this project was to further explore the potential utility of current TUS data for PA research. Specific goals of the analysis were to: (1) identify MET values for the detailed (4–6 digit) activity classification for US, UK and Poland; (2) report MET values for the 68-category MTUS cross-national historical harmonised activity classification and; (3) calculate daily PAL values for both the MTUS-68 and the detailed national UK, Polish and US activity lexicons based on weighted average activity frequencies and durations.

Together these elements further illustrate the utility of time use data for multinational studies of trends in energy expenditure and suggest more work is needed to document specific activities associated with paid and unpaid work and to measure intensity of self-selected activities for more accurate estimates of energy expenditure.

## Methods

The study involved five specific stages. First, we attached the MET values reported in the 2011 Ainsworth Compendium [54] to two new TUS activity coding lexicons; the Harmonised European Time Use Survey (HETUS) and MTUS as well as accessing the existing ATUS-MET Score linkage (stage 1). Next, we analysed national TUS data from the UK, US and Poland to identify daily activities across 24-h (stage 2), then calculated aggregate daily MET scores for the three activity coding lexicons using weighted average activity frequencies and durations for the harmonised MTUS activity categories based on underlying activity specific MET scores from HETUS and ATUS (stage 3). For stage 4, we carried out preliminary assessment work on the METs attributions by examining patterns of variation in daily METs by various socio-demographic characteristics (age, sex and education) and considering associations of aggregate daily METS with self-reported health status. Finally, we investigated any reductions in sample variability in METs using the less detailed MTUS-68 activity category compared with the more detailed ATUS and HETUS activity classifications (stage 5).

### Datasets

The MTUS includes nationally-representative TUS carried out in 25 countries dating from 1961. MTUS includes the Harmonised European Time Use Survey (HETUS) (Belgium, Germany, Estonia, France, Hungary, Slovenia, Finland, Sweden, UK and Norway), ATUS and other national-level TUS (Australia, Austria, Bulgaria, Canada, Denmark, Israel, Italy, Republic of Korea, Netherlands, South Africa, Spain and India) [24]. The data from the three countries reported here (as is the case for most of the surveys included in the MTUS) use population and day weights from the original national time use surveys.

As most of the recent European TUS follow the HETUS Guidelines [31] which provide pre-fieldwork guidelines for sampling, survey design and activity coding, we have a particularly well-matched HETUS compliant subset of newer European TUS with the MTUS. We used three nationally-representative TUS from the MTUS archive; US (2003–13), UK (2001) and Poland (2012), the latter two being HETUS compliant. Selected sample characteristics are provided in Additional file 1: Table S1.

### Procedure for assigning METs

As the starting point for ATUS, we followed the procedure recommended by Tudor-Locke and colleagues [39]. In general, we attached MET scores at the 6-digit level. When the main activity was transport, we assigned MET scores associated with the mode (e.g. walking, driving, cycling, public transport), whereas for paid work, we attached scores associated with the appropriate occupational group reported by Tudor-Locke and colleagues [39, 55]. Finally, for the very small proportion (< 0.1%) of activities not directly covered (e.g. job searching), we provided our own derived from the 2011 Compendium [45]. We grouped activities into eight broad categories for each of the 144 10 min (UK and Poland) or 288 5 min (US) TUD timeslots.

For the UK and Polish TUS datasets, we followed the same procedure as closely as possible, given the differences between the ATUS and the HETUS study designs and activity coding lexicons. We applied the codes used by Tudor-Locke and colleagues [39, 50, 55, 56] wherever we could identify exact activity equivalents and when required, produced our own codes by applying weighted combinations of Tudor-Locke and 2011 Compendium codes [45]. We also recoded the detailed 3-digit activity classifications into the simpler 2-digit 68-category MTUS harmonised classification for the three national TUS, assigning METs equivalents derived from the Tudor-Locke principles described above. Examples of assignments are listed in Additional file 2: Table S2; a full list of assignments for the three datasets is available on application to the corresponding author.

Whilst applying these procedures to the TUS data, we became concerned that the allocated METs may not adequately accommodate the wide range of PA intensity associated with some activities. One specific issue was the assignment of METs to different sorts of paid employment, as recommended by Tudor Locke and colleagues [50, 57]. Although calculating METs for sedentary jobs is quite straightforward, highly variable METs associated with manual occupations may be problematic and lead to overestimations [22].

### Estimating adherence to PA guidelines

PA is a multidimensional construct that incorporates frequency, intensity, duration and different types of activity, which can be reduced to a single variable reflecting the *total volume* of PA [58]. Whilst many studies have focussed on 10 min bouts of moderate to vigorous PA (MVPA) in line with the 2008 Physical Activity Guidelines for Americans (2008 PAG) [59], a number of studies show that light PA (LPA), moderate PA (MPA) and vigorous PA (VPA) all carry health benefits [60]. For this analysis, we estimated adherence to the 2008 PAG by aggregating time spent in activities from 3 - < 6 METs (MPA) and >/= 6 METs (VPA). Note that 150 min of MPA, 75 min of VPA activity (or an appropriately weighted combination of these) each week are considered adherent [59].

The single-day diaries used in this paper provide very conservative estimates. An individual falling substantially short of 150 min MPA on a random diary day may well still exceed it over the course of the week. We used the weekly cut-off to estimate the proportion meeting the PAG. Therefore, the surprisingly high level of compliance that emerges from even the single day statistics, once the full range of daily activities are included, is the basis for our conclusions that the MET’s attribution to diary activities should be reconsidered.

### Physical activity levels (PAL)

In each case, we estimated Physical Activity Levels (PAL) for each study participant [61]. PAL is equivalent to the aggregate mean daily METs (mdMETs) as the product of time spent in each designated activity category and the assigned MET score, producing a (sample weighted) sum across the entire 24-h day. This sum can be divided by 1440 to produce the PAL, a measure of energy expenditure expressed as a multiple of basal metabolic rate in common usage for comparative studies of energy expenditure. With a total of N distinct categories of activity:

$$ \mathrm{PAL}=\left(\ {\sum}_{n=1}^N\ \left({\mathrm{MET}}^{\mathrm{activity}\ \mathrm{n}}\ast \mathrm{DURATION}\ {\mathrm{in}\ \mathrm{minutes}}^{\mathrm{activity}\ \mathrm{n}}\right)\right)/1440 $$

## Results

PAL values in the three samples ranged from 1.60 to 1.74. The duration-weighting used in the PAL calculation implies that the means of the more and less detailed versions of the PAL for each survey should be similar (see Table 1). However, coefficients of variation may differ and indeed CVs ranged from 0.143 to 0.194 across countries and activity lexicons.

**Table 1:** Mean and variability of Physical Activity level (PAL)

	Mean	Standard deviation	Coefficient of variation	MTUS as % of original Coefficient of Variation
ATUS original	1.589	.291	0.194	
US MTUS 68-category	1.588	.227	0.155	80
UK HETUS original	1.626	.310	0.191	
UK MTUS 68-category	1.621	.237	0.146	77
Poland HETUS original	1.738	.337	0.183	
Poland MTUS 68-category	1.740	.270	0.143	78

### Effects of detail of classification on variability of PALs

Most respondents reported engaging in 20 to 25 different activities on any given day, and these are dispersed across the 68 MTUS categories. The ATUS has just under 400 6-digit activity categories, whilst the Polish and UK HETUS studies have approximately 250 and 300 4-digit categories, respectively. The 25 most frequently reported daily activities cover ~ 90% of all reported activities. Just two of these – sleeping and paid work – account for ~ 1000 of the 1440 min that constitute a day. Therefore, aggregating more detailed categories into the MTUS-68 should not result in any substantial changes to the activity groupings.

The coefficient of variation for the estimates from the much less detailed MTUS-68 category for each country is only around one fifth smaller (0.155, 0.146, 0.143 for the US, UK and Poland respectively) than the original classification systems (0.194, 0.191, 0.183).

### Effects of level of detail on relationship of PAL to sociodemographic characteristics

**Fig. 1:**
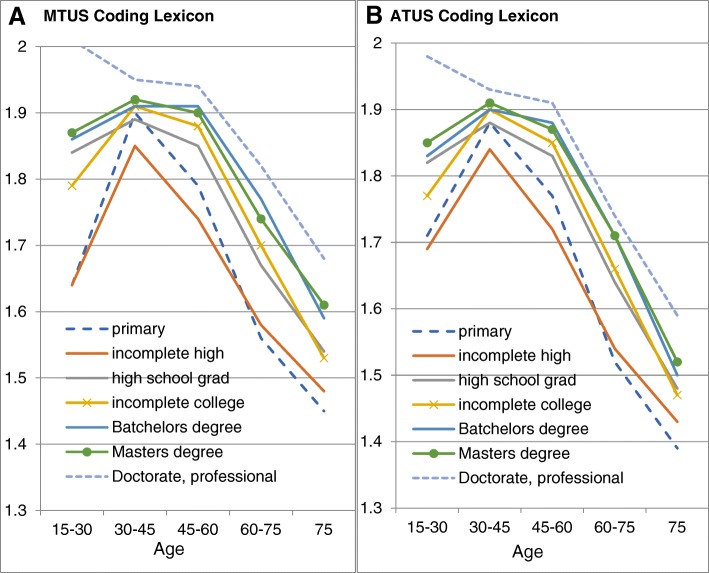
Physical Activity Level (PAL) estimates for men based on the MTUS harmonized activity codes (1**a**) and the American Time Use survey activity codes (1**b**) by age and education level in the USA

The choice of *more*- or *less*-detailed activity classifications has little impact on the relationship between age, educational attainment and the summary estimates of PA levels represented by PALs based on the more- and the less-detailed activity classifications. In the two panels of Fig. 1, the age distribution has the same inverted-U relation to PALs, and the generally monotonic association between educational attainment and PA is essentially unaffected by the choice between the two types of indicator. The only exception to the linear association of educational level with PA is amongst participants with a primary education only, who have PA levels intermediate between incomplete and complete high school, most likely because of the higher METs associated with manual occupations.

The UK and Polish results show similar inverted-U age distributions and monotomic positive relationships between education and PA. The pattern of sociodemographic variation in PALs for US women in both cases is similar to that for US men, and also to equivalent groups in the UK and Poland. The mean value for women in all cases is slightly lower than for men.

### The distribution of PA across the representative day

The central and most important advantage of employing the TUD approach to estimating EE and PALs is that all daily activities are represented (in terms of sequence, frequency and duration) across the 24-h day. This enables analysts to estimate time devoted to activities at different intensity levels on a five minute (US) or 10 min (UK and Poland) basis. Table 2 provides a complete picture of the general distribution of the days of the of the working-age adult populations in the three countries across eight broad categories of activity.

**Table 2:** Distribution of time spent in major activity categories

	Poland		UK		USA	
Minutes per day	Mean	St d	Mean	St d	Mean	St d
Sleep and personal care	624.6	166.9	630.3	124.1	619.1	144.1
Paid work	234.2	262	226.6	253	257.5	266
Unpaid work and education	153.3	138.2	145.8	134	124.5	147.5
Care of others	53.2	100.1	45.1	82.5	56.0	103.8
Exercise, walking, cycling	33.3	62.8	29.2	65.1	26.9	70.5
At home leisure	217.8	145.4	242.1	154	241.6	189.7
Away from home leisure	17.9	52.5	22.4	57.1	25.2	69.9
Travel	64.9	63.3	92.o	78.8	77.8	78.4
Other, missing	40.7	238	6.5	31.4	10.9	46.6
	1440	*n* = 48,996	1440	*n* = 11,993	1440	*n* = 53,046

In all three countries, the largest single category across the 24-h period is *sleep* and *personal care* (bathing, eating), while *paid* and *unpaid work* of various types, occupy ~ 8 h. By contrast *discretionary exercise* (e.g. sports, gym, swimming, cycling) occupy only ~ 30 min of the day in each case. The three countries differed most in time reported in work and travel, with Poles reporting less time in travel and US respondents reporting more time in paid work and less in unpaid work.

**Fig. 2:**
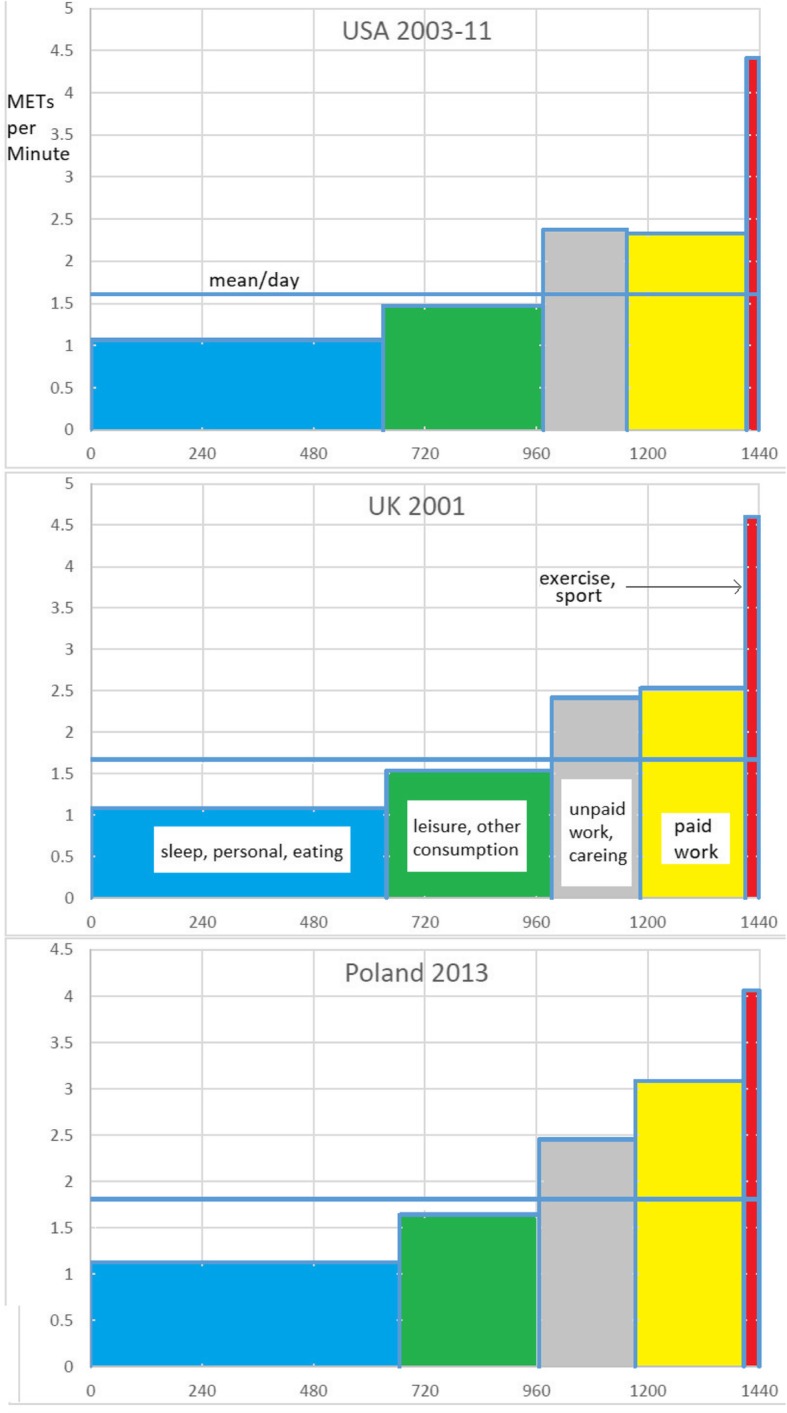
The daily balance of time and METs (national samples ages 20–59)

Table 3 shows the mean predicted levels of PA (METs) associated with each of the aggregate activity categories, following our assignment procedures. Combining the two tables, we arrive at the base-proportional histograms or ‘propograms’ (Fig. 2) which, in effect, decompose the PALs by the different types of activities out of which they are composed. In these base-proportional histograms, the horizontal axis represents the division of the day into activity domains, and the vertical axis represents the mean METs for each. The sum of the products of the width and the height of the various columns (i.e. the whole of the shaded area) establishes the total daily METs (i.e. PALs).

**Table 3:** Mean METs by activity

	Poland			UK			US		
	mean	St d	N	Mean	St d	N	Mean	St d	N
Sleep and personal care	1.1	0.07	47,610	1.08	0.07	11,993	1.06	0.08	53,044
Paid work	3.08	1.07	24,920	2.53	0.88	6070	2.33	0.69	30,083
Unpaid work & education	2.48	0.48	42,110	2.4	0.41	10,708	2.37	0.39	42,652
Care activities	2.38	0.47	21,297	2.47	0.48	5858	2.38	0.57	25,799
Exercise, walking, cycling	4.06	0.84	17,242	4.6	1.47	3663	4.41	1.38	13,595
At home leisure	1.41	0.06	46,247	1.41	0.11	11,732	1.41	0.12	49,937
Away from home leisure	1.69	0.26	10,743	1.62	0.29	3917	1.69	0.35	11,706
Travel	2.39	1.14	40,794	1.85	0.82	11,113	1.59	0.44	47,128
Other, missing	1.5	0	124	1.5	0	1634	1.3	0	6848

Note the very strong family similarity between the three national propograms. The great majority (> 90%) of MVPA is not derived from discretionary exercise, but from the two categories of paid, and unpaid work. In Poland, paid work is associated with relatively high mean METs levels.

### Ordering the events of the day by their activity intensity

Many different types of everyday activities (e.g. some paid work, housework, walking or cycling to work, gardening) provide opportunities for people to engage in MVPA. TUS data enable analysts to systematically identify the *duration* and *frequency* of all activities above specific target MET levels.

To ease the process of assessing respondents’ PAG compliance, the diary analyst may re-order the *chronological sequence* of the respondent’s activities, into an *activity EE sequence*, ordered from the highest to the lowest METs levels across the 24-h reporting period. The intensity-ordered daily sequence files may then be used to map time use into different PAEE categories, showing the proportion of respondents achieving specific METs levels for various durations across 24-h.

**Fig. 3:**
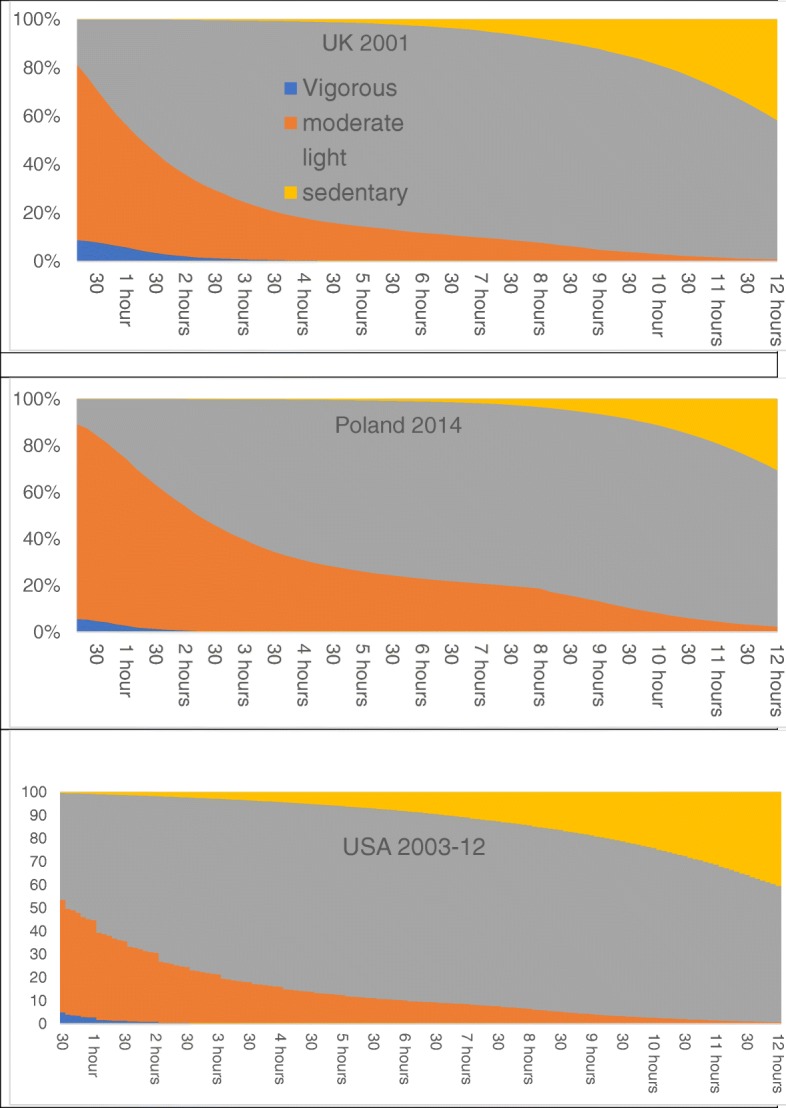
METograms: Percentage of subsamples reaching intensities for various durations (men and women aged 20–59)

Using the intensity-ordered sequence, we can then classify each of the 144/288 successive timeslots by the METs-estimated PAEE for each activity. Figure 3 provides ‘METograms’ that estimate the national populations’ distributions of PAEE sequences. Reading from the left, we see that, for the most active 10 min intervals of the day, more than 80% of Polish men and women, and just under 80% of UK adults report engaging in MVPA, whilst for US adults, the proportion is a little over 50%. This rate falls steeply for the UK and Polish populations, but less so for the US, leaving, at the 2 h 30 min point, approximately 25% of US, and 40% of UK and Polish adults engaged in MPA. Virtually none of the respondents engaged in 150 min of VPA on a single day.

The 2008 PAG [59] recommendation is a weekly > = 75 min VPA (> 6 METs), > = 150 min of MPA (3–6 METs) or an intermediate period with a weighted average of these (which we estimate on a 1-for-2 basis: 70 min VPA plus 10 min MPA, and so on.) The METs-ordered plot of the activities of a sample of single, randomly selected days in turn allow us to estimate a *minimum* value for the rate of compliance with PA Guidelines. (Clearly, non-compliant individuals on a given survey day may still achieve compliance during any of the six following days.)

Adherence to PA guidelines, on the single diary day, and based on vigorous activity alone was low, ranging from 2.6 to 6.1% (Table 4). A further 2% of the samples engaged in some vigorous activity on the diary day, and so met the guidelines through a mixture of moderate and vigorous activities. However, the great majority of respondents (95.4 in Poland, 92% in the UK and 95.1% in the US) did no vigorous activity at all during the diary day. Including those with no vigorous activity but some moderate (i.e. summing the first three rows of the table), single-day adherence levels rise substantially, ranging from 62.6% in Poland to 35.8% in the US. The relatively high level of single-day compliance, implying higher levels of weekly compliance, raises questions about the attributions of METs to activities.

**Table 4:** Proportion of sample with daily activity at various METs levels (men and women aged 20–59)

	Poland	UK	USA
> =75 min vigorous activity	2.6	6.1	3.0
weighted mix of moderate and vigorous	2.0	1.9	1.9
> = 150 min moderate activity	58.0	38.3	30.9
< 150 min of moderate	37.3	53.8	64.2
n =	48,996	11,993	53,046

## Discussion

Despite the evident advantages, relatively little validation work has been published on TUD-based approaches to estimating EE [62], perhaps because the major application has been in the social sciences, with little interest in physiological correlates of behaviour. The results of this project illustrate the feasibility of assigning MET scores by country to the harmonised 68-category MTUS data resource. This data linkage activity opens the possibility of conducting detailed analyses of changes in estimated PAEE in 25 countries across the six decades of data archived in the MTUS. It also provides further support for the general effort to link MET scores and TUS data [43, 49, 60]. The mean daily METs per minute approach to assessing PAEE has the merit of providing an appropriately balanced view of the distribution of people’s daily activity across all life domains, as in Fig. 2. This advantage is also leading to calls for greater use of 24-h physical activity recalls in health studies to examine associations between specific activities such as television viewing and health outcomes [38, 63].

We see immediately that discretionary PA accounts for <5% of daily activity, whereas sleep, paid work, and the remaining unpaid work and leisure time each account for nearly one third each. Similar results have been reported in past studies. For example, Dong et al. [43] report driving a car, office work, and watching TV as the top three activities responsible for energy expenditure in a large sample of US adults from 1992 to 94. The novel propogram and METogram graphics in this paper show the advantages of achieving a more comprehensive understanding of the extent and distribution of activities beyond the more frequently researched health domains of leisure time physical activity (LTPA) and discretionary PA. Relatively few studies have examined occupational physical activity (OPA) [22, 50, 64] and transport related PA [33, 65] using TUS data, and even fewer domestic production activities.

### Adherence to PA guidelines and global trends in PA

Estimates of the prevalence of adherence to PA guidelines obtained in this study were higher (35–64%) than those obtained from device based measurement with ambulatory cutpoints [8], despite the time use diary’s shorter observation window. The relatively higher estimates of adherence for Poland could be related to the larger proportion of manual labour remaining in the Polish economy.

There are two analytically distinct issues in relation to estimating adherence to PA guidelines. First are questions about the modality of measurement of daily activities, concerning both accuracy and comprehensiveness. Second are issues of associating MET levels with those activities.

Adherence estimates in the US range from around 5% based on accelerometer measurements using ambulatory cutpoints [8] to around 50% for studies using cutpoints based on a wider range of activities or multiple 24-h PA recalls [38]. An Australian TUS reported that 85% of a nationally representative sample of adults aged 65+ achieved 30+ minutes of MVPA based on estimates from two consecutive 24-h recalls [49]. Estimates of adherence based on some PA frequency questionnaires such as the International Physical Activity Questionnaire (IPAQ) range from ~ 55–90+ % [66].

In addition to the issues of measurement modality and associations of activities with METs levels, we also need to consider the focus of TUS on the purposes of time use (e.g. ‘work’) versus the physical activity focus on intensity and posture (e.g. ‘moderate’ or ‘sedentary’). These challenges could be addressed with additional questions concerning the purpose and intensity of activities involving PA, or time use focused data collection, although such questions increase respondent burden.

Single 24-h TUD measures provide valid estimates of group level activity patterns [67, 68]. However, estimating adherence to PA guidelines using TUD data requires either multiple-day diaries or statistical approaches incorporating responses to frequency questions or other covariates [36, 69]. In the present study, we report only single-day estimates of adherence. Further work on individual level measures of PA obtained from TUS could be useful for linkage studies of the associations between health outcomes and time-use.

Despite variation in estimates of adherence, many studies agree on temporal trends and national variation in the amount of PA estimated from various self-report instruments. PA levels have declined dramatically over time in the US and other countries [44, 70] and such declines have been observed in women as well as men [60]. Declining PA levels in multiple domains, including paid and unpaid work (such as household chores) were evident in the 1960s and even earlier in developed countries, but are also appearing in the developing economies of Brazil and China and, to a lesser extent, in India. These results suggest a potential association between obesity and levels of PA and support further comparative studies using the rich data of the MTUS harmonized data set. In the present study, prevalence of obesity and PA covary in the expected direction, but many more countries are needed for a robust ecological analysis.

### Challenges and limitations of MET score attribution to TUS activity lexicons

Although the TUD evidence alone is acceptably valid and reliable, some aspects of the MET scores attribution procedures require more investigation [22, 55]. These concerns relate primarily to jobs involving task variation at different MET levels. For example, desk-based jobs are relatively sedentary (1.3–1.5 METs), whilst manual labour jobs may range from sedentary (e.g. controlling traffic flow with a stop and slow sign at 2.0 METs) to vigorous (e.g. shovelling dirt at 6 METs).

Whilst the compendia [45, 52] provide laboratory evidence to support the MET scores associated with single generic tasks, the empirical evidence necessary to substantiate the proportional METs for different job tasks (and hence the appropriate weightings) is currently lacking. These issues of PA variation apply, to varying degrees, across the entire range of daily activities. The promising initial results of TUD-based MET estimations provide a strong case for further investment into research on the association of METs with the events of daily life. Greater detail concerning activities during paid and unpaid work could be particularly useful for improving estimates of PAL across different countries and time periods.

## Conclusions

TUD are a rich source of data concerning historical patterns of PA and SB. Further analysis of these data could help to generate hypotheses concerning trends in obesity and chronic conditions. Linking compendia of MET scores allows TUS data to be examined in units of EE, clarifying the potential for time use/health linkages. Based on our analyses of TUS from the US, UK and Poland, efforts to extend such linkages to other countries and to historical time use data dating back to the 1960s seems warranted. Special attention, however, will have to be given to the difficulty of disaggregating activity categories that include activities at different intensities.

The issues of measuring PAEE variation within periods devoted to paid and unpaid work of various types, merits attention in the form of new studies linking diary methods with continuous observation of PA through the day, such as the CAPTURE-24 project [29]. Diarists wearing accelerometers (and perhaps other devices, such as heart rate monitors and cameras) during their diary keeping periods, would provide appropriate evidence for the required recalibration of METs attributions to work and other activities.

If these problems can be solved, TUS could continue to grow as a valuable tool for surveillance of PALs in diverse countries, including those where financial and technical barriers limit the use of device-based measurement.

## Additional files

Additional file 1: **Table S1.** Unweighted sample characteristics. (DOCX 14 kb)

Additional file 2: **Table S2.** Sample METS assignments for MTUS. (DOCX 18 kb)

## Abbreviations

ATUS: American Time Use Survey
CV: Coefficient of Variation
EE: Energy Expenditure
HETUS: Harmonised European Time Use Survey
IPAQ: International Physical Activity Questionnaire
LPA: Light Physical Activity
LTPA: Leisure Time Physical Activity
mdMETs: Mean daily METS
MET: Metabolic Equivalent of Task
MPA: Moderate Physical Activity
MTUS: Multinational Time Use Study
MVPA: Moderate-to-Vigorous Physical Activity
OPA: Occupational Physical Activity
PA: Physical Activity
PAEE: Physical Activity Energy Expenditure
PAG: US Physical Activity Guidelines for Americans
PAL: Physical Activity Level
SB: Sedentary Behaviour
TUS: Time Use Survey/Study
VPA: Vigorous Physical Activity
