# Supplementary material for: Daily metabolic expenditures: estimates from US, UK and polish time-use data
Source: BMC Public Health. 2019 Jun 3;19(Suppl 2):453. doi: 10.1186/s12889-019-6762-9 (PMC6546617; doi:10.1186/s12889-019-6762-9)
Supplement: Supplementary file 1 — Table S1. Unweighted sample characteristics. (DOCX 14 kb) [file 12889_2019_6762_MOESM1_ESM.docx]

**Table S1 Unweighted sample characteristics**

**UK__________________________________ US___________________________________ Poland_______________________________**

**AGE 40.55 AGE 44.34 AGE 48.99**

**SEX(woman=2) 1.54 SEX(woman=2) 1.52 SEX(woman=2) 1.64**

**WORKHRS 37.78 WORKHRS 39.85 WORKHRS 19.03**

**DAY**

**Sunday 1 2837 Sunday 1 21186 Sunday 1 10955**

**Monday 2 2827 Monday 2 21179 Monday 2 10949**

**Tuesday 3 2846 Tuesday 3 21239 Tuesday 3 10951**

**Wednesday 4 2836 Wednesday 4 21306 Wednesday 4 10948**

**Thursday 5 2847 Thursday 5 21238 Thursday 5 10952**

**Friday 6 2806 Friday 6 20995 Friday 6 10948**

**Saturday 7 2825 Saturday 7 21199 Saturday 7 10953**

**19824 Total 148344 76656**

**EMPSTAT Economic activity**

**Econ active full time 1 7380 Employed at work 1 90111 Employed Full Time 1 25376**

**Econ active part time 2 2849 Employed absent 2 3714 Employed Part Time 2 2874**

**Econ active un'ployed 3 369 Unemployed on layoff 3 866 Employed hours unkn 3 8396**

**Econ inactive retired 4 3343 Unemployed looking 4 7853 Not in paid work 4 40010**

**Econ inactive fulltime 5 485 Not in labor force 5 45800 76656**

**Econ inactive looking 6 1076 148344**

**Econ inactive longtime 7 719**

**Econ inactive other 8 533**

**Adult not classified 9 211**

**Under 16yrs - inelig 10 2861**

**19824**

**UK__________________________________ US___________________________________ Poland_______________________________**

**EDCAT Highest qual.**

**Value Label**

**Degree level qual. 1 2210 Less than 1st grade 10 309 uncompleted secondary 1 14485**

**Higher edn below degree 2 1776 1st, 2nd, 3rd, or 4t 11 1050 completed secondary 2 45960**

**A levels, vocational 3 1791 5th or 6th grade 12 2109 above secondary education 3 16192**

**O levels, GCSE >=C 4 2626 7th or 8th grade 13 3964 -8 20**

**GCSE below grade C 5 525 9th grade 14 5234 76656**

**lower Qualification 6 294 10th grade 15 6064**

**Other qualification 7 573 11th grade 16 6314**

**Qualifications other 1 8 176 12th grade - no dipl. 17 2118**

**Qualifications other 2 9 95 High school graduate 20 3786**

**Qualifications other 3 10 32 High school graduate 21 40524**

**Qualifications other 4 11 65 Some college, no degree 30 25311**

**No qualifications 12 6800 Associate degree - o 31 5702**

**Eligible - No answer 13 1 Associate degree - a 32 6097**

**Under 16yrs - inelig 14 2861 Bachelor's degree (B 40 25774**

**19824 Master's degree (MA,MSc) 41 10147**

**Professional school 42 1959**

**Doctoral degree (PhD) 43 1882**

**148344**
