# Supplementary material for: Daily metabolic expenditures: estimates from US, UK and polish time-use data
Source: BMC Public Health. 2019 Jun 3;19(Suppl 2):453. doi: 10.1186/s12889-019-6762-9 (PMC6546617; doi:10.1186/s12889-019-6762-9)
Supplement: Supplementary file 2 — Table S2. Sample METS assignments for MTUS. (DOCX 18 kb) [file 12889_2019_6762_MOESM2_ESM.docx]

| **Table S2: Sample METS assignments for MTUS** | | |  | | |
| --- | --- | --- | --- | --- | --- |
|  | Mean METs |  |  | Mean METs |  |
| sleep and naps | 0.92 |  | wash, dress, care for self | 2.10 |  |
| meals at work or school | 1.50 |  | other meals or snacks | 1.50 |  |
| paid work - main job (not at home) | 3.11 |  | paid work at home | 3.02 |  |
| second or other job not at home | 1.50 |  | unpaid work for household income | 4.30 |  |
| work breaks | 1.50 |  | other time at workplace | 1.50 |  |
| look for work | 1.50 |  | regular schooling, education | 1.82 |  |
| homework | 1.80 |  | leisure/other education or training | 2.30 |  |
| food preparation, cooking | 2.16 |  | set table, wash/put away dishes | 2.32 |  |
| cleaning | 3.03 |  | Laundry, ironing, clothes repair | 2.07 |  |
| home/vehicle maintenance/improvement | 3.65 |  | other domestic work | 2.13 |  |
| purchase goods | 2.24 |  | consume personal care services | 1.18 |  |
| consume other services | 1.68 |  | pet care (not walk dog) | 2.72 |  |
| physical, medical child care | 2.66 |  | teach, help with homework | 2.64 |  |
| read to, talk or play with child | 2.64 |  | supervise accompany, other childcare | 1.65 |  |
| adult care | 2.46 |  | voluntary, civic, organisation acts | 1.89 |  |
| worship and religious activity | 1.89 |  | general out-of-home leisure | 1.63 |  |
| attend sporting event | 1.50 |  | cinema, theatre, opera, concert | 1.80 |  |
| other public event, venue | 2.20 |  | restaurant, café, bar, pub | 1.50 |  |
| party, reception, social, gambling | 2.07 |  | general sport or exercise | 6.51 |  |
| walking | 3.80 |  | cycling | 8.00 |  |
| other out-of-doors recreation | 3.04 |  | gardening/forage, hunt, fish | 3.68 |  |
| walk dogs | 3.80 |  | receive or visit friends | 1.50 |  |
| conversation (in person, phone) | 1.50 |  | other in-home social, games | 1.64 |  |
| general indoor leisure | 1.50 |  | artistic or musical activity | 2.10 |  |
| written correspondence | 1.50 |  | knit, crafts or hobbies | 2.33 |  |
| relax, think, do nothing | 1.50 |  | read | 1.50 |  |
| listen to music, CD, audio book | 1.38 |  | listen to radio | 1.15 |  |
| watch TV, DVD, video | 1.33 |  | play computer games | 1.50 |  |
| send e-mail, internet, computing | 1.50 |  | just mode of recorded travel | 1.85 |  |
| travel to or from work | 2.23 |  | education-related travel | 2.41 |  |
| travel for voluntary/civic/religious | 2.59 |  | child/adult care-related travel | 2.29 |  |
| shopping, personal, household travel | 2.55 |  | traveling for other purposes | 2.41 |  |
| no recorded activity | 1.50 |  |  |  |  |
